# An Empirical Analysis of the Influence of Volleyball Elective Course on Students' Physical Health Based on Digital Image

**DOI:** 10.1155/2022/9229912

**Published:** 2022-03-24

**Authors:** Shiwei Wang

**Affiliations:** Chengdu Sport University, Chengdu, Sichuan 610041, China

## Abstract

In recent years, due to the continuous improvement of the national economic level and the increasing academic burden of students' main courses, students' physical health problems (e.g., obesity, vision, and lumbar spine) have become more and more serious, which urgently needs the attention of relevant departments of national education and parents. This paper will use digital image technology to create a physical parameter measurement system and use literature, comparative analysis, and other research methods to analyze the impact of volleyball elective courses on students' physical health. Firstly, this paper explains the theory of image processing technology and analyzes the parameters of human body scientifically; secondly, it detects the physical parameters of human body in digital images and also designs an image recognition system; finally, through experimental analysis, the accuracy of identifying key points of images is relatively high. After the system is adopted, the error of the measurement index is small. After the training of human body indexes, the effect of volleyball can be effectively improved.

## 1. Introduction

Students are the future pillars of a country and the hope and seeds of a country. However, with the rapid development of science and technology, students enjoy the convenience of life by using mobile phones, computers, and other electronic devices for a long time. Long-term inactivity (or a small amount of exercise) leads to students' insufficient physical fitness and gradual decline in physical fitness, which is contrary to our concept of “sunshine sports.” In order to cultivate students' idea of “lifelong physical education and physical fitness,” this paper selects volleyball elective course to test the specific role of students' health, using digital image technology to analyze and study, discuss and help students to make a better fitness plan suitable for individuals from various angles and factors, truly make people-oriented, provide effective scientific basis and health guidance suggestions, and strive to improve the physique of all students. Reference [1] introduces the basis of digital image, image enhancement in spatial domain and frequency domain. Literature [2] uses a class of algorithms to determine the local similarity between structured data sets as the basis of pattern recognition and image processing. Literature [3] investigates and analyzes digital image recording methods and reviews common standards and guidelines. Reference [4] combines artificial neural network with digital image to analyze grain quality. Reference [5] explains the use of digital image related theory and algorithm structure in processing algorithms and applications. Literature [6] used experiments to find out the comprehensive effects of physical fitness and psychological variables on abdominal strength, grip and psychological skills of volleyball players after training. Literature [7] showed the training effect of explosive leg force and shoulder strength of volleyball players after training by comparing the situation after training in Kupas. Literature [8] shows that the application of stair teaching method in volleyball elective courses in colleges and universities can effectively improve students' comprehensive volleyball ability. Literature [9] uses algorithms to detect the motion features based on body trajectory clustering and aggregation in volleyball and collects the required motion data. Literature [10] introduces the effective improvement of intensity modified leisure volleyball on the health signs and physical fitness of men aged 25–55. Literature [11] discusses the influence of four-person soft volleyball teaching practice, which is beneficial to college students' physical and mental health. Literature [12] reviews and analyzes the research on students' mastery of health-related fitness knowledge so as to clear up misunderstandings. Literature [13] investigated the attitudes and physical activity behaviors of professionals before participating in sports, health, and leisure services. Literature [14] evaluates the body composition, health awareness, and cardiopulmonary health of female college students after compulsory physical education class. Literature [15] studies the theoretical and empirical analysis of the influence of age and exercise on physical activity. The article introduces the image processing technology to carry on the movement recognition to the volleyball movement; the human body physique characteristic and the scientific movement training through the high recognition rate can be scientifically analyzed. Analysis of the human body constitution in the article shows that, after training, the body shape is more increased than before, and the overall weight shows a downward trend. Volleyball elective course has different degrees of improvement on each student's physical fitness, and to achieve the best results, so as to strengthen the diversified teaching practice.

## 2. Theoretical Basis

### 2.1. Research Methodology

This paper mainly adopts the following three methods to study, analyze, and compare the theory and data in order to ensure the reliability, authenticity, and effectiveness of the results and avoid adverse effects caused by other unexpected factors.

#### 2.1.1. Literature Research Method [16]

This paper refers to a large number of websites and documents such as CNKI, China Academic Journal Network, and Baidu Academic, which provides scientific theoretical basis for the study of students' physical health. In addition, this paper also consulted a large number of books, periodicals, lectures, conferences, and magazines related to physical education quality courses, physical education teaching quality reform, volleyball elective courses, and so on, in order to deeply understand and grasp the research content of this topic from all aspects and angles.

#### 2.1.2. Data Statistics [17]

As the name implies, this method is to collect and sort out all kinds of data obtained from experimental tests so as to facilitate the analysis and discussion of the results. This method will use Excel 2016 software [18] and SPSS 17.0 tool [19] to collect various indicators for statistical steps.

#### 2.1.3. Comparative Analysis [20]

According to different categories and requirements, the physical health indicators of students obtained after the experimental test are compared and analyzed, such as body shape indicators and physical fitness indicators. Finally, the conclusion is summarized.

### 2.2. Digital Images

#### 2.2.1. Introduction of Concepts

**Figure 1:**
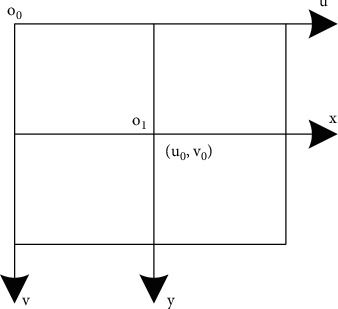
The coordinates of the image.

A digital image [21] is one of the images, represented by a two-dimensional array [22], and the basic digital unit is pixel points [23], which can be directly processed by a computer. The other analog image cannot be processed by computer unless it is digitally converted. The image format used in this paper is mainly JPEG format, which is mainstream and can save space by compressing storage, and basically all shooting equipment can support it. In this paper, according to the brand effect and use effect, we finally decide to use a certain Sony device to complete the shooting work. As shown in Figure 1, it is about the representation coordinate system of pixels on the image.

#### 2.2.2. Instructions for Image Acquisition

In this paper, firstly, the two-dimensional plane image is taken to ensure the high quality of the image quality, and the image parameter standard is unified, and the two-dimensional plane image is converted into the parameter data of the three-dimensional index.

#### 2.2.3. Image Processing Techniques

**Figure 2:**
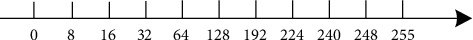
Gray value.

**Figure 3:**
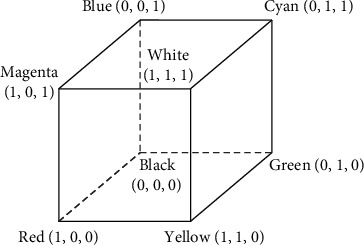
RGB color space.

(*1) Image Grayscale* [24]. It is a general popular processing technique that relies on RGB [25] color proportions to operate. It can improve the speed of image processing, transform the original color image with too large pixels into gray image with fewer pixels, and reduce the burden of image processing. As shown in Figures 2 and 3, it is a spatial representation of the relationship between gray value number axis and RGB color scale.

As shown below, there are several conversion methods related to image grayscale. According to the actual needs and convenience, we chose the fifth method to effectively separate human parameters from surrounding environmental parameters.

Method 1: floating-point algorithm.

(1) $$\begin{array}{c} Gray=R*0.3+G*0.59+B*0.11. \end{array}$$

Method 2: integer method.

(2) $$\begin{array}{c} Gray=\left(R*30+G*0.59+B*11\right)/100. \end{array}$$

Method 3: shift algorithm.

(3) $$\begin{array}{c} Gray=\left(R*76+G*151+B*28\right)\gg 8. \end{array}$$

Method 4: average method.

(4) $$\begin{array}{c} Gray=\left(R+G+B\right)/3. \end{array}$$

Method 5: only take the green method.

(5) $$\begin{array}{c} Gray=G. \end{array}$$

Suppose a grayscale photo of *M*×*N* size; list all possible grayscale values *z*_*i*_, *i*=0,1,2, ⋯, *L* − 1. We can get the probability of gray level *z*_*k*_ in this photo:

(6) $$\begin{array}{c} p\left(z_{k}\right)=\frac{n_{k}}{MN}, \end{array}$$

(7) $$\begin{array}{c} \overset{L-1}{\underset{k=0}{\sum }}p\left(z_{k}\right)=1. \end{array}$$

From formulas (6) and (7), the average grayscale, grayscale variance, and *n*-step distance can be obtained:

(8) $$\begin{array}{c} m=\overset{L-1}{\underset{k=0}{\sum }}z_{k}p\left(z_{k}\right), \end{array}$$

(9) $$\begin{array}{c} \sigma^{2}=\overset{L-1}{\underset{k=0}{\sum }}{\left(z_{k}-m\right)}^{2}p\left(z_{k}\right), \end{array}$$

(10) $$\begin{array}{c} \mu_{n}\left(z\right)=\overset{L-1}{\underset{k=0}{\sum }}{\left(z_{k}-m\right)}^{n}p\left(z_{k}\right). \end{array}$$

*(2) Image Arithmetic Operation*. If the image is disturbed by Gaussian noise, superposition operation is performed:

(11) $$\begin{array}{c} s\left(x,y\right)=f\left(x,y\right)+g\left(x,y\right). \end{array}$$

Image enhancement operations are

(12) $$\begin{array}{c} d\left(x,y\right)=f\left(x,y\right)-g\left(x,y\right). \end{array}$$

Select a special image block:

(13) $$\begin{array}{c} p\left(x,y\right)=f\left(x,y\right)\times g\left(x,y\right). \end{array}$$

**Figure 4:**
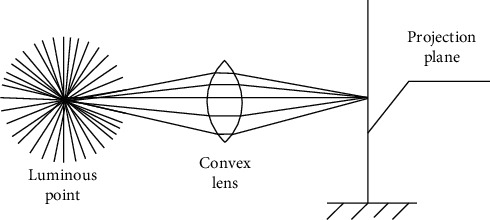
Principle of lens shadow generation.

Because the shot lens is affected by optical characteristics, shadows will occur. The lens principle is as shown in Figure 4.

We perform shadow correction of the image:

(14) $$\begin{array}{c} v\left(x,y\right)=\frac{f\left(x,y\right)}{g\left(x,y\right)}. \end{array}$$

*(3) Geometric Space Transformation*. It can improve the spatial relationship of coordinates between pixels, where (*v*, *w*) = original pixel coordinates and (*x*, *y*) = transformed pixel coordinates.

(15) $$\begin{array}{c} \left(x,y\right)=T\left\{\left(v,w\right)\right\}. \end{array}$$

*(4) Image Transformation*. The goal is to transform into a more convenient form by calculation, and then transform back, which can effectively improve the processing efficiency.

Unified formula of transformation is

(16) $$\begin{array}{c} T\left(u,v\right)=\overset{M-1}{\underset{x=0}{\sum }}\overset{N-1}{\underset{y=0}{\sum }}f\left(x,y\right)r\left(x,y,u,v\right). \end{array}$$

Corresponding inverse transformation unified formula is

(17) $$\begin{array}{c} f\left(x,y\right)=\overset{M-1}{\underset{u=0}{\sum }}\overset{N-1}{\underset{v=0}{\sum }}T\left(u,v\right)s\left(x,y,u,v\right). \end{array}$$

For example, the process of filtering to remove interference sources is illustrated:

(18) $$\begin{array}{c} r\left(x,y,u,v\right)=e^{-j2\pi \left(ux/M+vy/N\right)}, \end{array}$$

(19) $$\begin{array}{c} s\left(x,y,u,v\right)=\frac{1}{MN}e^{j2\pi \left(ux/M+vy/N\right)}. \end{array}$$

Formulas (18) and (19) are substituted into formulas (16) and (17), respectively, and the result of transformation is as follows:

(20) $$\begin{array}{c} T\left(u,v\right)=\overset{M-1}{\underset{x=0}{\sum }}\overset{N-1}{\underset{y=0}{\sum }}f\left(x,y\right)e^{-j2\pi \left(ux/M+vy/N\right)}, \end{array}$$

(21) $$\begin{array}{c} f\left(x,y\right)=\frac{1}{MN}\overset{M-1}{\underset{u=0}{\sum }}\overset{N-1}{\underset{v=0}{\sum }}T\left(u,v\right)e^{j2\pi \left(ux/M+vy/N\right)}. \end{array}$$

**Figure 5:**
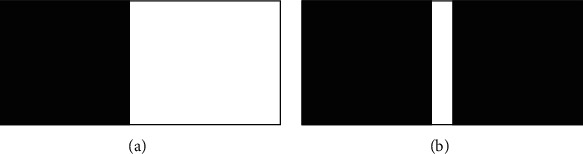
Edge of gray image. (a) Step-like. (b) Pulsatile.

*(5) Image Edge Detection*. As shown in Figure 5, it is an introduction to different types of edge shapes.

**Figure 6:**
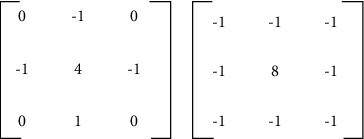
Laplacian operator template.

The principle of image edge detection is to identify the change of gray value in image edge. Based on Laplacian operator, its template is as Figure 6.

The relevant formula is as follows:

(22) $$\begin{array}{c} \nabla^{2}f\left(x,y\right)=\frac{\partial^{2}f\left(x,y\right)}{\partial x^{2}}+\frac{\partial^{2}f\left(x,y\right)}{\partial y^{2}}, \end{array}$$

(23) $$\begin{array}{c} \nabla^{2}f\left(x,y\right)=f\left(x+1,y\right)+f\left(x-1,y\right)+f\left(x,y+1\right)+f\left(x,y-1\right)-4f\left(x+1,y\right). \end{array}$$

*(6) Image Binarization*. If we want to detect our target object from the initial image, we use this method.

(24) $$\begin{array}{c} g\left(x,y\right)=\left\{\begin{array}{c} 0\left(Gray\;value\;is\;less\;than\;threshold\left(T\right)\right)\\ 255\left(Gray\;value\;is\;greater\;than\;threshold\left(T\right)\right) \end{array}.\right. \end{array}$$

In formula (24), *threshold* (*T*) is the judgment threshold, and the corresponding optimal value is generally obtained by continuously comparing performance parameters in experimental comparison.

### 2.3. Parameter Indicators

#### 2.3.1. Confirmation of Human Body Size Proportion

Using digital image technology to measure the human body, the obtained data is calculated in equal proportion with the defined human body size ratio, and the original real human body data is restored. The defined size ratio is shown in Table 1.

The formula model for obtaining data from the formula of human cross section model is as follows:

(25) $$\begin{array}{c} AX^{2n}+BX^{n}Y^{n}+CY^{n}=Z^{2n}, \end{array}$$

(26) $$\begin{array}{c} Y=R\times \;\sin\;\theta \times \alpha . \end{array}$$

#### 2.3.2. Unit Conversion

Because the unit of image is pixel unit, to convert it into actual human body parameters, it is necessary to convert pixel unit into actual centimeter unit. This conversion is based on 100 cm as a unit length, and the conversion formula is as follows:

(27) $$\begin{array}{c} L_{\left(Actual\right)}=L_{\left(Pixel\right)}*\left(100/L_{\left(Ruler\right)}\right)cm. \end{array}$$

In the second part of the article, the related research methods are explained, and then the digital image concept, acquisition, and processing technology are explained, and then the human body parameter characteristic parameters are explained proportionally. The second part gives an effective explanation of the basic research work of the article, which has good scientific significance.

## 3. Physical Parameter Detection Based on Digital Images

### 3.1. Analysis of the Characteristics of Volleyball Elective Courses

Volleyball elective course has antagonism across the net, competition and skill, which can stimulate students to learn volleyball elective course more passionately and enhance their physique. Volleyball class requires students to be skilled in volleyball basic skills, solid and stable core strength, know the rules of sports venues and referee rules of volleyball matches, skillfully use their own physical strength and skills, and be able to react quickly to volleyball, and meet the requirements of eyesight and self-control. Therefore, in volleyball elective training, physical education teachers only think about how to enhance students' physical fitness reasonably and effectively in order to meet the requirements of students' activities inside and outside class. As shown in Table 2, a key explanation about volleyball elective course is presented.

### 3.2. Analysis of Students' Physique

In recent years, with the rapid development of the world, the rising power of science and technology has brought many changes in life. These changes make people's lives more convenient, but also bring many troubles to people, among which physical health problems are particularly prominent. Due to the long-term academic pressure, students' spare time is occupied by homework and electronic equipment, and only a few students take physical exercise, which leads to the gradual decline of students' physical health and presents a subhealth state, which deserves the attention of relevant state departments and parents, and time should be given to encourage extracurricular activities.

When we test, we will use various indicators (e.g., height, weight, and vital capacity) to reflect the basic health status of a student according to the national test standards and make reasonable analysis and suggestions.

### 3.3. Design and Implementation of the System

#### 3.3.1. Functional Design of the System

**Figure 7:**
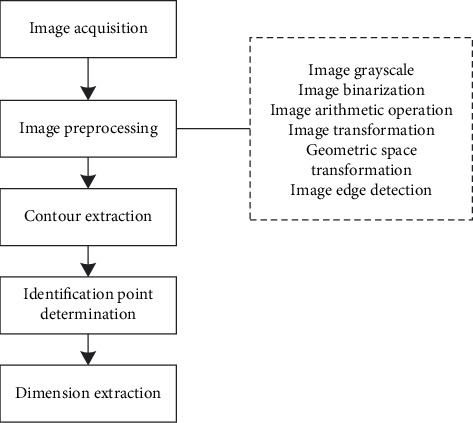
System operation flowchart.

The system designed in this paper is a tool for measuring human body in experimental test, adopting advanced digital image technology, using shooting equipment to record the image of human body, and using related image processing technology to preprocess the image. It includes a series of operations such as image grayscale, image arithmetic operation, image binarization, image transformation, image edge detection, and so on, and truly restores and converts the real data of human body as the data reference of the experiment, which will liberate the tedious and repeated work in the experiment and automatically obtain human body parameters. A flowchart of the image processing of the system is shown in Figure 7.

#### 3.3.2. Implementation of the System

1. LabVIEW 2013 software in Windows 10 operating system is selected as the development language. This programming language is more concise than the traditional programming language; there are more practical functional modules, more suitable for this system design needs.
2. After the size extraction, the system will use Excel 2016 software and SPSS 17.0 tools to collect various indicators for statistical steps so that the data can be saved more conveniently and reflected intuitively, which is convenient for us to analyze.
3. Realize the design drawing.

**Figure 8:**
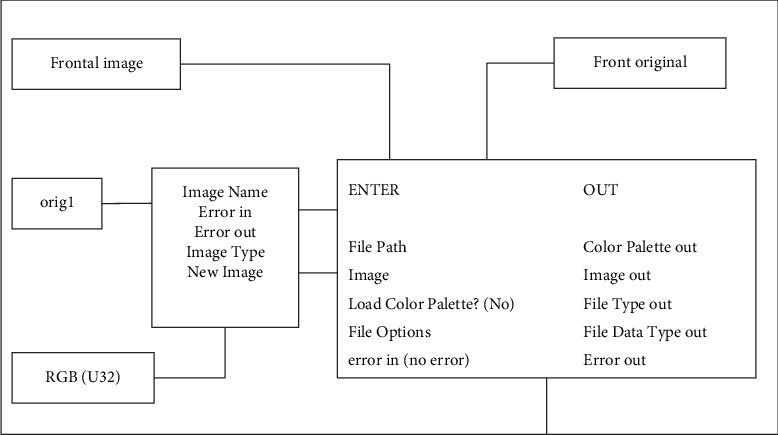
Design of reading function.

First, temporarily create a memory location to store the images we need to wait for processing. The functional design of the image reading part is shown in Figure 8.

The types of images are shown in Table 3.

**Figure 9:**
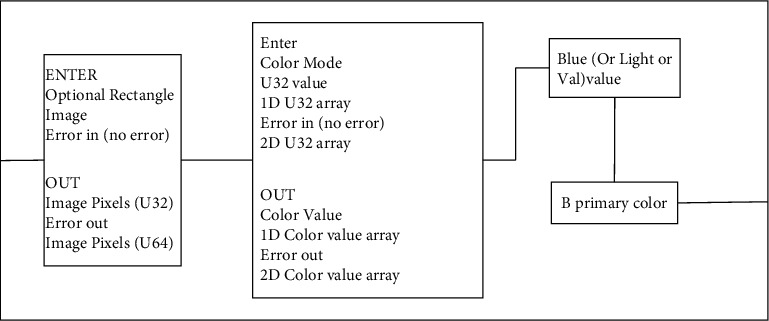
Design of gray level extraction function.

Second, the purpose of this program is to obtain the B primary color (color image), and the functional design diagram of extracting grayscale is shown in Figure 9.

Among them, the pattern of color is shown in Table 4.

Third, let the value of B primary color become the gray value of each pixel; we can use MATLAB code to achieve gray image. As shown in Algorithm 1, it is about the process realized by MATLAB code, and we choose three methods to run it.

**Figure 10:**
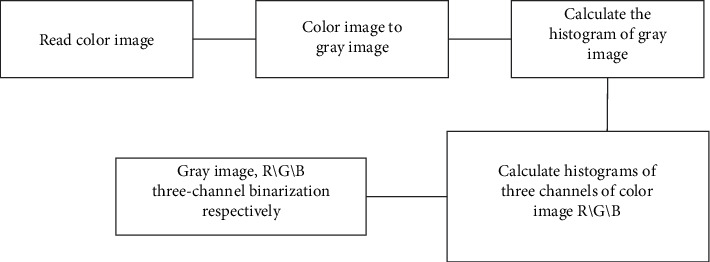
Image binarization flow.

Fourthly, the acquired gray image is processed, and the binarization of the image is carried out by MATLAB, as shown in Figure 10.

**Figure 11:**
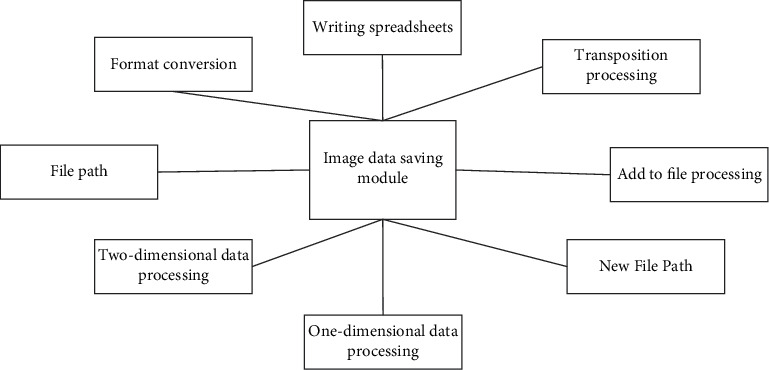
Design of image data saving function.

Fifthly, all kinds of data are uniformly converted into strings and then stored in corresponding files. The image data saving function design diagram is shown in Figure 11.

## 4. Experimental Data Analysis

### 4.1. Subjects of the Study

This paper takes the influence of volleyball elective course on students' physical health as the research object and makes a comparative study on the changes of students' physical health before and after volleyball elective course. Because the student group includes kindergarten, primary school, junior high school, high school, and university, in order to avoid the influence of physical development on students' health, we choose full-time freshmen in a university as the research object and randomly select 100 students (half male and half female) to participate in the physical health test. The test is divided into three stages. The first stage is that all people have not participated in volleyball elective courses for the first time, the second stage is that all people have been tested for the second time after two years of volleyball exercise with the same intensity (they can no longer participate in other physical exercises), and the third stage is the third test after stopping training for one year. Due to space constraints, we only choose to show part of the test data.

### 4.2. Analysis of Image Processing

#### 4.2.1. Image Scanning Determination

**Figure 12:**
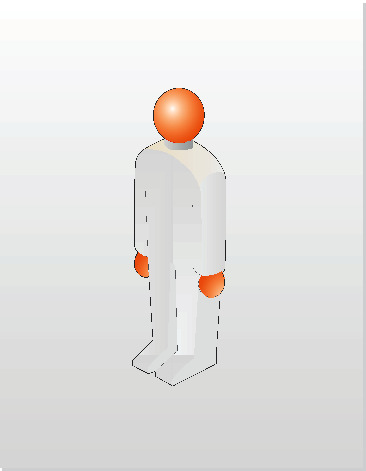
Calibration drawing of human body.

**Figure 13:**
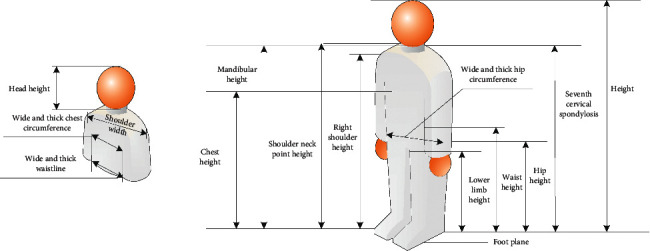
Key points of human body scanning.

1. An example of a scan line determination diagram of a human body is shown in Figure 12.
2. The confirmation of key points in human body scanning is shown in Figure 13.

#### 4.2.2. Scanning Recognition Accuracy Analysis

**Figure 14:**
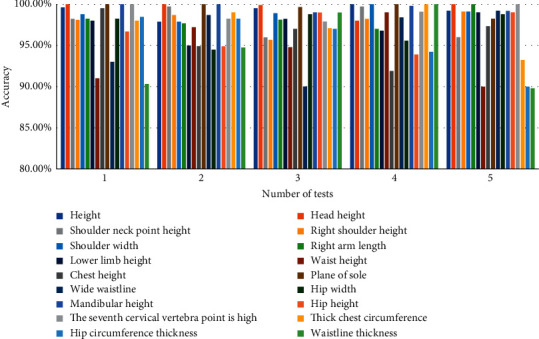
Recognition accuracy.

When processing digital images, it is very important to accurately confirm key points. Therefore, we analyzed the data of whether the digital image can accurately identify the key points in the human body and conducted five tests, respectively, as shown in Figure 14.

**Figure 15:**
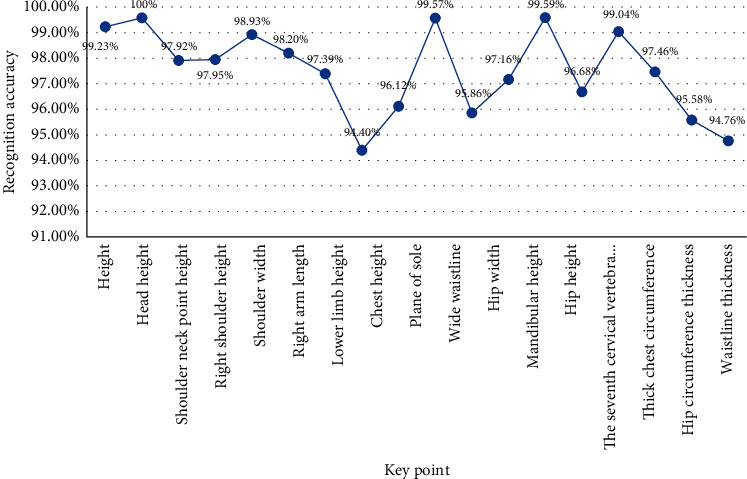
Average accuracy.

We calculate the average of the test results of five times and show the results in the form of line chart, as shown in Figure 15.

*Analysis*. We can find that the accuracy of digital image recognition is over 94%; the highest can reach 100%. The experimental results show that the accuracy of key points in image recognition is very high, which can meet the needs of the experiment.

### 4.3. Data Reliability Analysis

The data detected by our system still need to be verified and the reliability of the data is analyzed. Only by comparing the error between the data tested by the system and the data measured by the real system can we judge that the measured data of our system is true, reliable, and effective. Otherwise, the system cannot be adopted and needs to be redesigned and improved. In this trial run of the system, we considered labor cost, time cost, and other reasons, and only selected ten students for this data test, and adopted anonymous methods to protect students' privacy. However, in order to ensure the accuracy of the manual test results, we promise to repeat the test three times to select the average value.

As shown in Table 5, it is about the comparison of male students' height and arm span length.

As shown in Table 6, it is about the comparison of female students' height and arm span length.

As shown in Table 7, it is a comparison of the data of male students' measurements (chest circumference, waist circumference, and hip circumference).

As shown in Table 8, it is a comparison of the measurements (chest circumference, waist circumference, and hip circumference) of female students.

*Conclusion*. Through the comparison of the above table data, we can find that the error of each measurement index is very small, basically within the range of 0.2 cm, which can meet the needs of students' physical health detection in our experiment. After the system is adopted, we can find that its efficiency will be far greater than that of manual measurement.

### 4.4. Comparative Analysis of Data

Because the data of 100 students was tested, the data was too huge, so in the comparative analysis and display of the data here, we only randomly intercepted a small number of students' data (which was inconsistent with the student sample number when testing the system, and renumbered it) and adopted anonymous methods to protect students' privacy.

#### 4.4.1. Comparative Analysis of Basic Physical Indicators

(1) We compare and analyze the data of height and arm span of male students and female students, respectively, and give the corresponding line chart, which is convenient to feel the specific situation of the data more intuitively. As shown in Table 9, it is an analysis of the basic physical indicators of boys.

**Figure 16:**
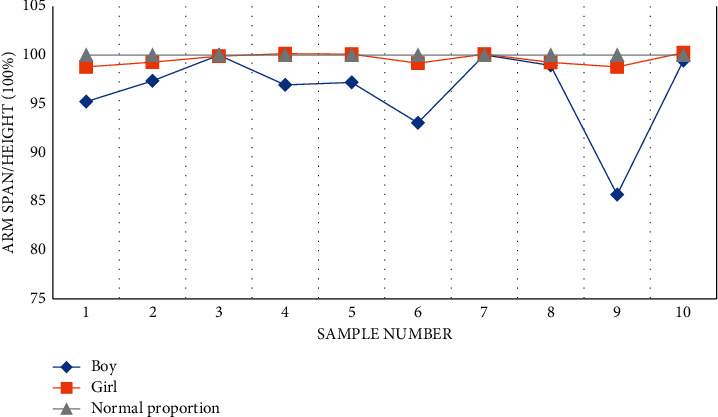
Basic index diagram of the body.

As shown in Figure 16, it is a broken line expression about the basic indicators of the body.

*Analysis*. By counting the basic physical indicators of all boys and girls (the data of 100 students are not shown here), we can find that the height range of boys is about (170, 180) and that of girls is about (150, 165). On the whole, there is little difference in height, among which some are prominent in height and some are short in height. After calculation, we find that the height and arm length of the students are basically 1 : 1, which belongs to the normal range of ordinary people. In some cases, the arm span is greater than the height. According to the trend analysis of line chart, there is a positive correlation between students' arm span and height.

**Figure 17:**
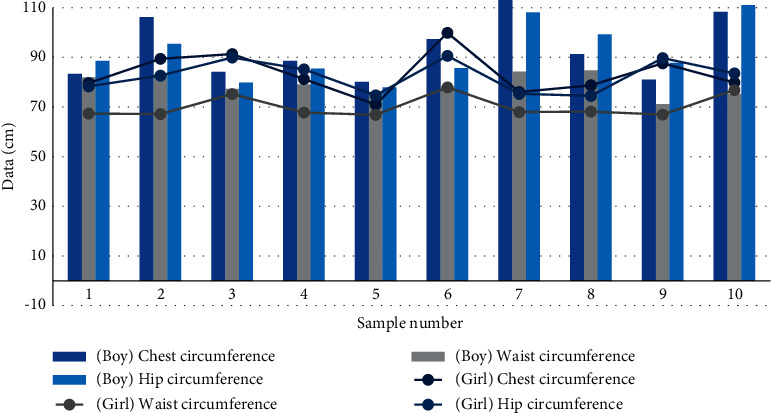
Body circumference index map.

(2) As shown in Figure 17, it is a broken line expression form about the body circumference index (part) of boys and girls.

*Analysis*. According to the above figure, we can find that no matter boys or girls, their individual circumference development is different, among which the chest circumference is the biggest difference, the hip circumference is the second, and the waist circumference curve is relatively gentle. On the whole, the fluctuation range of their respective circumference data curves is about 0 cm to 20 cm.

#### 4.4.2. Comparative Analysis of Body Shape Indicators

In this part, we mainly compare three stages, BMI index, and vital capacity of male and female students.

**Figure 18:**
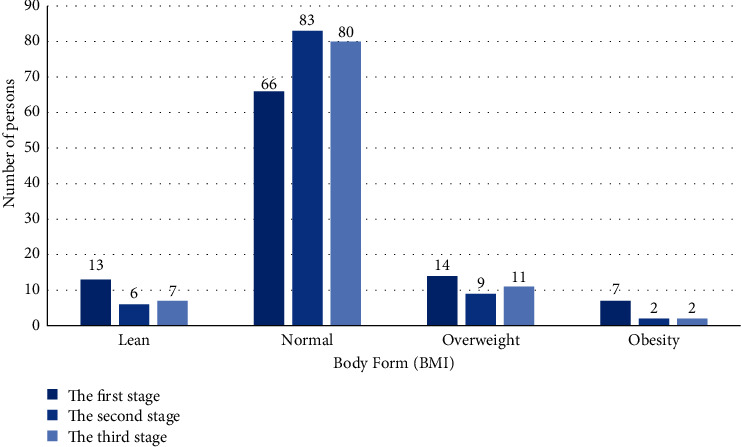
BMI exponential distribution in three stages.

(1) As shown in Figure 18, it is a comparative analysis chart of BMI index of all students in three stages.

**Figure 19:**
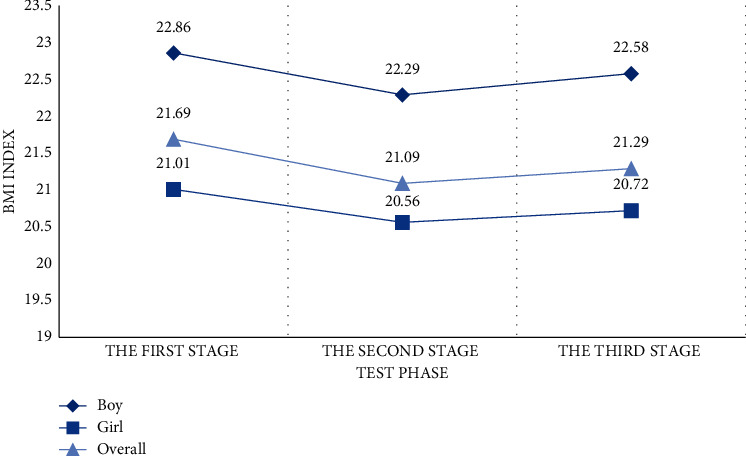
BMI mean distribution.

As shown in Figure 19, it is a comparative analysis of the average BMI index of boys and girls.

*Analysis*. Before the first stage of training, students with abnormal body shape accounted for 34% of the total number, among which lean and overweight were the most. After two years of volleyball elective training, we can find that 83% of people have normal body shape, which is 25.76% higher than the previous number. The number of students who are thin, overweight, and obese has dropped significantly. After stopping training for one year, the number of students with normal shape decreased by about 3.61%, and the number of obese students did not increase again. We found that the weight of the subjects showed a downward trend as a whole.

**Figure 20:**
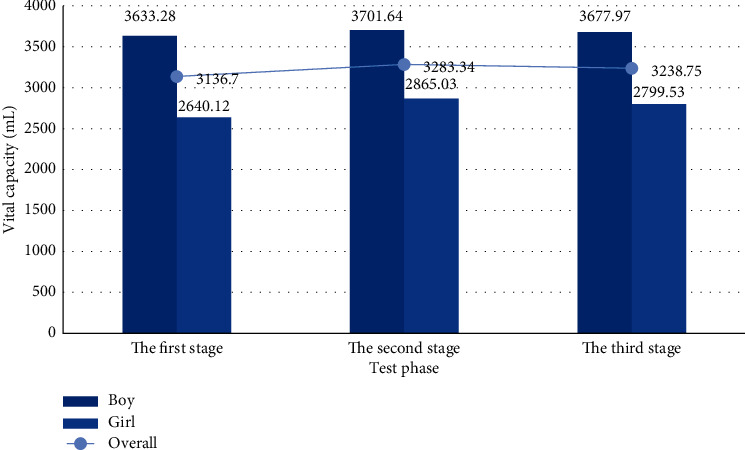
Distribution of mean vital capacity.

(2) As shown in Figure 20, it is about the average distribution of vital capacity of boys and girls.

*Analysis*. From the figure, we can find that the overall mean change of vital capacity of both boys and girls shows a small upward trend. In the second stage, the vital capacity of students increased the most. Although it decreased to a certain extent in the third stage, it can be seen that the cardiopulmonary respiratory system of students improved obviously.

#### 4.4.3. Comparative Analysis of Physical Fitness Indicators

In this part, we divided the data into two tests, which are before and after the volleyball elective class. As shown in Tables 10 and 11, it is about the average analysis and comparison of physical fitness indicators of male and female students.

*Analysis*. According to Tables 10 and 11, we can find that no matter whether boys or girls, after training, the training effect of five items is obvious, and the physical fitness indicators of students have a certain increase. This shows that volleyball elective course can effectively help students improve their physical quality and sports ability.

## 5. Conclusion

Whether the change of human body constitution can grow healthily is a key issue for the state to pay attention to. The purpose of this study is to empirically analyze the impact of volleyball elective courses on students' physical health, to illustrate the role of volleyball elective courses by using theory and data, to prove that volleyball elective courses can effectively improve students' physical fitness, and to cultivate students' enthusiasm and interest in sports so as to guide students to love sports and match their physical fitness with their learning quality. This paper analyzes the influence of volleyball on students' physique by using digital image processing technology. Through the experiment, we can get the conclusion that after the system is adopted, the error of the measurement index is small. For boys and girls, the development of three circumferences is different, the difference of chest circumference is the largest, the difference of hip circumference is the second, and the curve of waist circumference is relatively gentle. After training, the body shape is larger than before, and the overall weight shows a downward trend.

Volleyball optional courses have different degrees of improvement on each student's physical fitness, and to achieve the best results, we need more diversified teaching experience. The focus of future work should be tailored according to their different physical qualities, and individualized teaching programs should be formulated and implemented in order to achieve the scientific goals proposed in the article.

## Figures and Tables

**Algorithm 1:**
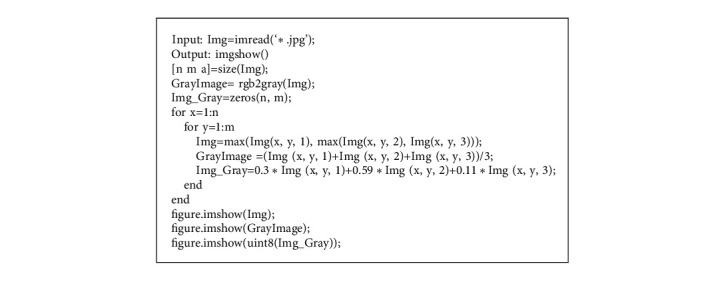
Gray image by MATLAB code.

**Table 1:** Size ratio of each part of the human body.

Position	Boy	Girl
Height	1.00	1.00
Eyes	0.94	0.93
Neck	0.84	0.86
Shoulder	0.82	0.81
Axillary	0.75	0.75
Chest circumference	0.72	0.72
Diaphragm	0.70	0.70
Elbow	0.63	0.63
Waist circumference	0.61	0.63
Hip circumference	0.53	0.53
Crotch	0.47	0.47
Thigh	0.41	0.41
Knee	0.26	0.26

**Table 2:** Characteristics of volleyball elective courses.

Name	Volleyball
Basic technology	Step, double pad, serve
Specialized technology	Application of tactical skills
Physical quality	Speed, strength, agility

**Table 3:** Table of image types.

Type	Size per pixel
Gray scale (U8)	8 bits (unsigned, standard monochrome)
Gray scale (16)	16 bits (signed)
Gray scale (SGL)	32 bits (floating point type)
Gray scale (U16)	16 bits (unsigned, standard monochrome)
Composite (CSG)	2 *∗* 32 bits (floating-point type)
RGB (U32)	32 bits (red, green, blue, transparency)
HSL (U32)	32 bits (chromaticity, saturation, brightness, transparency)
RGB (U64)	64 bits (red, green, blue, transparency)

**Table 4:** Color pattern table.

RGB	Default value
HSL	Conversion to HSV
HSV	Conversion to HSV
HSI	Conversion to HSV

**Table 5:** Data table of male students' height and arm span (unit: cm).

Sample number	Height (automatic)	Height (salary)	Arm extension (automatic)	Arm extension (artificial)
1	173.77	173.56	167.52	167.36
2	180.01	179.98	175.85	175.45
3	175.33	175.02	175.85	175.24
4	176.37	176.01	172.21	171.98
5	169.03	168.96	165.44	164.95
6	174.81	174.25	168.04	167.36
7	187.30	187.02	172.73	172.56
8	167.52	166.95	149.30	149.40
9	174.81	174.25	163.88	163.75
10	173.77	173.54	167.52	167.39

**Table 6:** Data table of height and arm span of girls (unit: cm).

Sample number	Height (automatic)	Height (salary)	Arm extension (automatic)	Arm extension (artificial)
1	160.23	159.98	158.67	157.39
2	161.25	160.89	160.55	159.68
3	159.45	159.35	159.33	159.54
4	152.27	152.45	152.48	152.98
5	153.68	153.65	153.83	153.22
6	164.72	164.54	164.17	163.95
7	150.73	150.05	150.26	150.95
8	169.59	169.32	168.65	168.06
9	157.49	157.24	157.30	157.65
10	158.23	158.24	158.67	158.66

**Table 7:** Data sheet of male students' measurements (unit: cm).

Sample number	Bra (automatic)	Bra (artificial)	Waist circumference (automatic)	Waist circumference (salary)	Hip circumference (automatic)	Hip circumference (artificial)
1	83.41	83.44	81.99	82.01	88.63	88.94
2	106.22	105.84	83.22	83.71	95.52	95.47
3	84.16	84.28	77.32	77.53	79.91	80.11
4	88.66	88.49	79.21	79.33	85.54	85.31
5	80.13	80.09	75.88	75.69	77.92	77.87
6	97.40	97.25	78.36	78.17	85.67	85.58
7	113.11	112.89	84.30	84.31	108.15	108.04
8	91.31	91.48	84.78	84.59	99.31	99.21
9	81.09	80.83	71.21	71.06	87.88	87.64
10	108.34	108.29	75.35	75.24	111.13	110.97

**Table 8:** Data sheet of girls' measurements (unit: cm).

Sample number	Bra (automatic)	Bra (artificial)	Waist circumference (automatic)	Waist circumference (salary)	Hip circumference (automatic)	Hip circumference (artificial)
1	79.69	79.70	67.42	67.39	78.35	78.28
2	89.41	89.45	67.18	67.09	82.65	82.59
3	91.34	91.28	75.21	75.63	89.95	89.87
4	81.27	81.09	67.78	67.81	85.26	85.28
5	70.98	71.13	66.85	66.79	74.73	74.80
6	99.89	98.04	77.93	77.96	90.65	90.81
7	76.02	76.19	67.95	67.85	75.35	75.41
8	78.77	78.85	68.22	68.16	74.53	74.68
9	87.60	87.59	66.96	66.83	89.78	89.64
10	79.83	79.93	76.85	77.06	83.59	83.62

**Table 9:** Basic body indicators (unit: cm).

Boy	Arm span/height (100%)	Girl	Arm span/height (100%)
1	95.25	1	98.80
2	97.36	2	99.30
3	100.01	3	99.90
4	96.95	4	100.16
5	97.22	5	100.11
6	93.05	6	99.20
7	100.04	7	100.12
8	98.95	8	99.27
9	85.71	9	98.80
10	99.44	10	100.26

**Table 10:** Comparison of physical fitness indexes of boys.

Project	Before training	After training
50 m	7.92 ± 6.77	7.31 ± 8.20
Standing long jump	216.30 ± 16.09	246.97 ± 13.12
Sitting body flexion	8.62 ± 3.3	8.35 ± 6.12
Pull-up	7.66 ± 4.03	10 ± 3.10
1000 m	238.10 ± 18.55	240.13 ± 19.01

**Table 11:** Comparison of physical fitness indexes of girls.

Project	Before training	After training
50 m	8.55 ± 10.22	8.10 ± 10.89
Standing long jump	165.05 ± 37.50	179.68 ± 41.07
Sitting body flexion	11.80 ± 6	10.2 ± 6.56
1 min sit-up	30.45 ± 7.55	37.52 ± 7.87
800 m	241.90 ± 13.30	241.41 ± 14.53

## Data Availability

The experimental data used to support the findings of this study are available from the author upon request.

## References

[1] Sunoj S., Subhashree S. N., Dharani S. (2018). Sunflower floral dimension measurements using digital image processing. *Computers and Electronics in Agriculture*.

[2] Barnea D. I., Silverman H. F. (2009). A class of algorithms for fast digital image registration[J]. *IEEE Transactions on Computers*.

[3] Cheddad A., Condell J., Curran K., Mc Kevitt P. (2010). Digital image steganography: survey and analysis of current methods. *Signal Processing*.

[4] Nakano N., Ohsumi A., Yoshida K. (2001). Identification of physical parameters of cantilevered beams from noisy vibration data. *Transactions of the Japan Cociety of Mechanical Engineers Series C*.

[5] Pitas I. (2000). Digital image processing algorithms and applications[J]. *IEEE Signal Processing Magazine*.

[6] Subathra P., Elango M., Subramani A. (2021). Combined effect of parcourse training and mental training on selected physical and psychological variables among volleyball players[J]. *Journal of Information and Computational Science*.

[7] Subathra P., Elango M., Subramani A. (2021). Effect of parcourse training on leg explosive power and shoulder strength among volleyball players[J]. *International Journal of Analytical and Experimental Modal Analysis*.

[8] Chen S., Wanglili, Chen Z. (2018). An empirical study on the application of stair -type teaching method in volleyball elective course in colleges and universities[J]. *Sports Science and Technology Literature Bulletin*.

[9] Kubota E., Suzuki T., Honda M., Ikenaga T. (2016). Action detection of volleyball using features based on clustering of body trajectories[J]. *The Journal of the Institute of Image Electronics Engineers of Japan*.

[10] Vasić G., Trajković N., Mačak D. (2021). Intensity-modified recreational volleyball training improves health markers and physical fitness in 25-55-year-old men. *BioMed Research International*.

[11] Chen J., Chen S. (2006). Study of the effect of four people’s soft volleyball on the physical and mental health of college students[J]. *Journal of Jilin Institute of Physical Education*.

[12] Xiaofen D. K., Harrison L., Chen L., Xiang P., Castro Piñero J. (2009). An analysis of research on student health-related fitness knowledge in K–16 physical education programs[J]. *Journal of Teaching in Physical Education*.

[13] Huddleston S., Mertesdorf J., Araki K. (2002). Physical activity behavior and attitudes toward involvement among physical education, health, and leisure services pre-professionals[J]. *College Student Journal*.

[14] Konczos C., Bognár J., Szakály Z., Barthalos I., Simon I., Oláh Z. (2012). Health awareness, motor performance and physical activity of female university students. *Biomedical Human Kinetics*.

[15] Klein T., Becker S. (2012). Age and exercise: a theoretical and empirical analysis of the effect of age and generation on physical activity. *Journal of Public Health*.

[16] Dutta S., Pal S. K., Mukhopadhyay S., Sen R. (2013). Application of digital image processing in tool condition monitoring: a review. *CIRP Journal of Manufacturing Science and Technology*.

[17] Yakin S., Hasanuddin T., Kurniati N. (2021). Application of content based image retrieval in digital image search system. *Bulletin of Electrical Engineering and Informatics*.

[18] Chen G. B., Sun Z., Zhang L. (2020). Road identification algorithm for remote sensing images based on wavelet transform and recursive operator. *IEEE Access*.

[19] Zhou Y., Huang Y. (2018). On the application of the practice method in the volleyball teaching in public sports course——taking the elective courses of public volleyball in a college in shaanxi province as an example.% the application of “replacing training with competition” in the teaching of public sports volleyball pad [J]. *Sports Boutique (Academic Edition)*.

[20] Park K. Y., 김동원 D., 어영숙 Y., 서보원 B. (2010). Impact of sitting volleyball program on the isokinetic muscular strength and mental health of women with disabilities. *Korean Journal of Physical, Multiple, & Health Disabilities*.

[21] Zhang S., Mao H. (2021). Optimization analysis of tennis players’ physical fitness index based on data mining and mobile computing. *Wireless Communications and Mobile Computing*.

[22] Dai X., Li S. (2021). Volleyball data analysis system and method based on machine learning. *Wireless Communications and Mobile Computing*.

[23] Watson A., Biese K., Kliethermes S. A., Post E., Mcguine T. (2021). Impact of in-season injury on quality of life and sleep duration in female youth volleyball athletes: a prospective study of 2073 players[J]. *British Journal of Sports Medicine*.

[24] Ji H., Zheng C. (2021). The influence of physical exercise on college students’ mental health and social adaptability from the cognitive perspective[J]. *Work*.

[25] Ormsbee M. J., Kinsey A. W., Chong M., Heather S., Friedman (2013). The influence of high intensity interval training on the salivary cortisol response to a psychological stressor and mood state in non-sedentary college students[J]. *Journal of Exercise Physiology Online*.

